# Book Reviews

**Published:** 1922-11

**Authors:** 


### BOOK REVIEWS

Orthodontic Impression and Casts—By Herbert A. Pullen, D.M.D.

This little manual of ninety pages incorporates in a very concise and readable manner the best known instructions as to the art of making plaster models for records and study of cases. This work which Dr. E. H. Angel perfected, Dr. Pullen has carried out more perfectly than almost any other teacher of the work of orthodontia. The technic he presents is standard, and is the result of much study and the best thoughts of the orthodontic schools. The author presents his plan of impression taking as well as model making. The various steps are clearly described so that the procedures should not be difficult for students and practitioners to follow. Good impressions and models properly made and prepared for study and preservation are invaluable aids in orthodontic practice. Dr. Pullen has done his work so well that no one can make a critical comment on his book. It certainly gives one all the essentials in a remarkable clear-cut and complete exposition in this admirable little book. The book is printed in the usual manner of the P. Blakistone's Son & Co. publishing house, and the eighty illustrations are a fine exposition of the printer's art. It is printed for $1.50, and any one interested in this subject will be well paid for a carefull study of the book.

---

Dental and Oral Radiography—By James David McCoy, M.S., D.D.S.

Dr. McCoy has revised the text and illustrations in his new edition of his book with the intention of making this little manual render a full exposition of the principles more easily comprehended by students and operators. This subject, while new in its large and growing field, has reached a degree of standardization, at least, of the theory involved. It is necessary that one should carefully read and study this book to be able to keep up to present-day practices. Dr. McCoy has presented the update theories and practices of the radiographic art so clearly that no one should have difficulty in practicing the modern methods.

The large development of radiography as an accepted and required method of diagnosis in handling diseased teeth is absolutely esential in dental practice. It is surprising how dependent we are on our knowledge of unseen pathologic conditions. We almost dare not to venture an opinion without support from the radiographic findings. We have received incalculable help from this source, and we have to acknowledge our indebtedness to Dr. McCoy for the fine work he has done, and which he here again has splendidly given us an up-to-date study of this problem, from both the technical and scientific study of this great problem. We shall study this new book with increasing pleasure and profit, I am sure. It is the work of a thorough and thoughtful student and his long experience will strengthen our confidence in practical diagnoses. The book is well printed and illustrated, by the C. V. Mosby Co., of St. Louis. We cordially commend it to the profession as the most up-to-date manual on Dental Radiography.

